# 3α-Hydr­oxy-*ent*-atis-16-en-14-one

**DOI:** 10.1107/S1600536809026002

**Published:** 2009-07-11

**Authors:** Huan Wang, Kai-Bei Yu, Li-Sheng Ding, Xiao-Duo Luo, Xiao-Feng Zhang

**Affiliations:** a North-West Institute of Plateau Biology, Chinese Academy of Sciences, Xining 810001, People’s Republic of China; b Chengdu Institute of Organic Chemistry, Chinese Academy of Sciences, Chengdu 610041, People’s Republic of China; c Chengdu Institute of Biology, Chinese Academy of Sciences, Chengdu 610041, People’s Republic of China; d State Key Laboratory of Phytochemistry and Plant Resources in West China, Kunming Institute of Botany, Chinese Academy of Sciences, Kuming 650204, People’s Republic of China

## Abstract

The title compound, C_20_H_30_O_2_, is an *ent*-atisane diterpenoid which was isolated from the roots of *Euphorbia kansuensis*. The mol­ecule contains five six-membered rings, among which three six-membered rings of the bicyclo­[2.2.2]octane unit adopt boat conformations and two cyclo­hexane rings adopt chair conformations. In the crystal structure, mol­ecules are connected by inter­molecular O—H⋯O hydrogen bonds, forming zigzag chains propagating parallel to [001].

## Related literature

For applications of the roots of *Euphorbia kansuensis*, see: Zhao & Zhao (1992 ▶). For related structures, see: Lal *et al.* (1990 ▶); He *et al.* (2008 ▶).
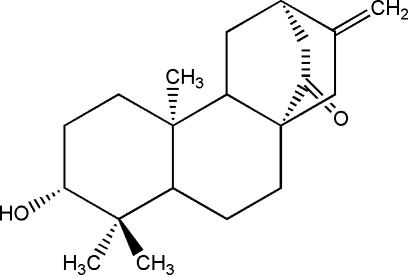


## Experimental

### 

#### Crystal data

C_20_H_30_O_2_
*M* *_r_* = 302.44
Orthorhombic, 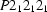

*a* = 7.310 (1) Å
*b* = 12.346 (2) Å
*c* = 18.431 (3) Å
*V* = 1663.4 (4) Å^3^
*Z* = 4
Mo *K*α radiation
μ = 0.08 mm^−1^
*T* = 285 K
0.54 × 0.38 × 0.30 mm

#### Data collection

Siemens P4 diffractometer
Absorption correction: none
2435 measured reflections
1744 independent reflections
1240 reflections with *I* > 2σ(*I*)
*R*_int_ = 0.020
3 standard reflections every 97 reflections intensity decay: 2.8%

#### Refinement

*R*[*F* ^2^ > 2σ(*F* ^2^)] = 0.040
*wR*(*F* ^2^) = 0.076
*S* = 0.96
1744 reflections
207 parameters
1 restraint
H atoms treated by a mixture of independent and constrained refinement
Δρ_max_ = 0.12 e Å^−3^
Δρ_min_ = −0.13 e Å^−3^

### 

Data collection: *XSCANS* (Siemens, 1994 ▶); cell refinement: *XSCANS*; data reduction: *SHELXTL* (Sheldrick, 2008 ▶); program(s) used to solve structure: *SHELXS97* (Sheldrick, 2008 ▶); program(s) used to refine structure: *SHELXL97* (Sheldrick, 2008 ▶); molecular graphics: *SHELXTL*; software used to prepare material for publication: *SHELXTL*.

## Supplementary Material

Crystal structure: contains datablocks I, global. DOI: 10.1107/S1600536809026002/xu2540sup1.cif

Structure factors: contains datablocks I. DOI: 10.1107/S1600536809026002/xu2540Isup2.hkl

Additional supplementary materials:  crystallographic information; 3D view; checkCIF report

## Figures and Tables

**Table 1:** Hydrogen-bond geometry (Å, °)

*D*—H⋯*A*	*D*—H	H⋯*A*	*D*⋯*A*	*D*—H⋯*A*
O1—H1*O*⋯O2^i^	0.813 (10)	2.110 (11)	2.922 (3)	178 (3)

Symmetry code: (i) 
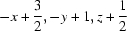
.

**Fig. 1.**
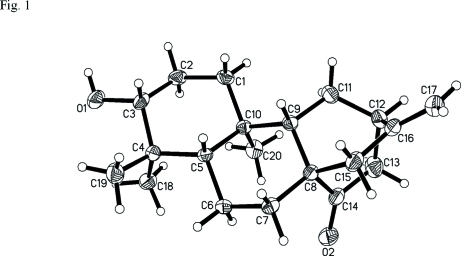
The molecular structure of the title compound, with atom labels and 50% probability displacement ellipsoids for non-H atoms.

**Fig. 2.**
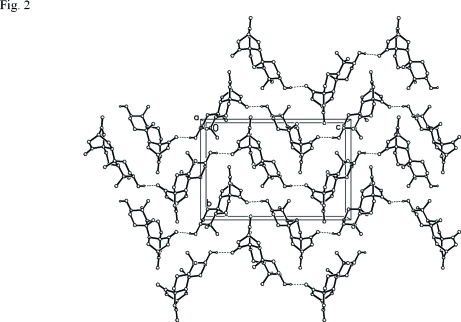
A partial packing diagram of the title compound. Hydrogen bonds are shown as dashed lines.

## supplementary crystallographic information

### Comment

*Euphorbia**kansuensis* Proch. (Euphorbiaceae) is distributed mainly in the west of China. As a Tibetan medicine, the roots of this plant have been used as pyretolysis, cholagogue, apocenosis and purgative (Zhao & Zhao, 1992). Our investigation of the roots of this plant led to the isolation of the title compound. The compound has been reported previously and its structure was postulated from spectroscopic methods (He *et al.*, 2008). In order to further confirm the spatial structure, a crystal structure analysis has been undertaken.

The molecular structure (Fig. 1) contains five six-membered rings (A, atoms C1–C5/C10; B, C5–C10; C, C8/C9/C11–C14; D, C8/C12–C16 and E, C8/C9/C11/C12/C15/C16). Rings A and B adopt a chair conformation, while rings C, D and E of the bicyclo-[2.2.2]-octane adopt boat conformations. The A/B and B/E ring junctions are trans-fused, but B/C is cis-fused. In the crystal structure, the molecules are linked by intermolecular O—H···O hydrogen bonds, forming the one-dimensional structure (Fig. 2).

### Experimental

The air-dried roots of E. kansuensis (15 kg) were extracted with 85% EtOH (2 × 30 l) at 358 K for 2 h and then evaporated in vacuo. The residue suspended in water was extracted with CHCl_3_. The CHCl_3_ extract (180 g) was subjected to Si-gel CC using solvents of increasing polarity from petroleum ether through EtOAc to afford 15 fractions (F1-F15). Fraction F7 was further separated by RP-18 CC using MeOH-H_2_O (68:32) to give the title compound (18 mg), and further crystallized at room temperature from MeOH to afford prisms. The analytical NMR data of (I) are in accordance with the reference (He et al., 2008)

### Refinement

H atoms were positioned geometrically (C—H = 0.93–0.98 Å and O—H = 0.81 Å). H atoms bonded to C atoms were refined as riding, with *U*_iso_(H) = 1.2*U*_eq_(C). The absolute configuration could not be determined from the X-ray analysis because of the absence of strong anomalous scatterers. Friedel pairs were therefore merged before refinement. However, the absolute configuration may be suggested on a biogenetic basis (Lal *et al.*, 1990; He *et al.*, 2008).

### Crystal data

**Table:**

C_20_H_30_O_2_	*F*(000) = 664
*M**_r_* = 302.44	*D*_x_ = 1.208 Mg m^−^^3^
Orthorhombic, *P*2_1_2_1_2_1_	Mo *K*α radiation, λ = 0.71073 Å
Hall symbol: P 2ac 2ab	Cell parameters from 28 reflections
*a* = 7.310 (1) Å	θ = 2.8–13.3°
*b* = 12.346 (2) Å	µ = 0.08 mm^−^^1^
*c* = 18.431 (3) Å	*T* = 285 K
*V* = 1663.4 (4) Å^3^	Prism, colourless
*Z* = 4	0.54 × 0.38 × 0.30 mm

### Data collection

**Table:**

Siemens P4 diffractometer	*R*_int_ = 0.020
Radiation source: normal-focus sealed tube	θ_max_ = 25.3°, θ_min_ = 2.0°
graphite	*h* = 0→8
ω scans	*k* = 0→14
2435 measured reflections	*l* = −1→22
1744 independent reflections	3 standard reflections every 97 reflections
1240 reflections with *I* > 2σ(*I*)	intensity decay: 2.8%

### Refinement

**Table:**

Refinement on *F*^2^	Secondary atom site location: difference Fourier map
Least-squares matrix: full	Hydrogen site location: inferred from neighbouring sites
*R*[*F*^2^ > 2σ(*F*^2^)] = 0.040	H atoms treated by a mixture of independent and constrained refinement
*wR*(*F*^2^) = 0.076	*w* = 1/[σ^2^(*F*_o_^2^) + (0.031*P*)^2^] where *P* = (*F*_o_^2^ + 2*F*_c_^2^)/3
*S* = 0.96	(Δ/σ)_max_ < 0.001
1744 reflections	Δρ_max_ = 0.12 e Å^−^^3^
207 parameters	Δρ_min_ = −0.13 e Å^−^^3^
1 restraint	Extinction correction: *SHELXL97* (Sheldrick, 2008), Fc^*^=kFc[1+0.001xFc^2^λ^3^/sin(2θ)]^-1/4^
Primary atom site location: structure-invariant direct methods	Extinction coefficient: 0.0228 (17)

### Special details

**Table:**

Geometry. All e.s.d.'s (except the e.s.d. in the dihedral angle between two l.s. planes) are estimated using the full covariance matrix. The cell e.s.d.'s are taken into account individually in the estimation of e.s.d.'s in distances, angles and torsion angles; correlations between e.s.d.'s in cell parameters are only used when they are defined by crystal symmetry. An approximate (isotropic) treatment of cell e.s.d.'s is used for estimating e.s.d.'s involving l.s. planes.
Refinement. Refinement of *F*^2^ against ALL reflections. The weighted *R*-factor *wR* and goodness of fit *S* are based on *F*^2^, conventional *R*-factors *R* are based on *F*, with *F* set to zero for negative *F*^2^. The threshold expression of *F*^2^ > σ(*F*^2^) is used only for calculating *R*-factors(gt) *etc*. and is not relevant to the choice of reflections for refinement. *R*-factors based on *F*^2^ are statistically about twice as large as those based on *F*, and *R*- factors based on ALL data will be even larger.

### Fractional atomic coordinates and isotropic or equivalent isotropic displacement parameters (Å^2^)

**Table:**

	*x*	*y*	*z*	*U*_iso_*/*U*_eq_	
O1	0.6384 (3)	0.66962 (17)	0.56057 (11)	0.0535 (6)	
O2	0.6926 (3)	0.34246 (17)	0.20401 (9)	0.0586 (7)	
C1	0.8688 (4)	0.4212 (2)	0.48422 (14)	0.0411 (7)	
H1A	0.8136	0.3692	0.5170	0.049*	
H1B	0.9969	0.4023	0.4789	0.049*	
C2	0.8547 (4)	0.5341 (2)	0.51763 (16)	0.0448 (8)	
H2A	0.9139	0.5344	0.5648	0.054*	
H2B	0.9173	0.5859	0.4869	0.054*	
C3	0.6573 (4)	0.5667 (2)	0.52594 (14)	0.0378 (7)	
H3	0.5982	0.5127	0.5571	0.045*	
C4	0.5518 (4)	0.5697 (2)	0.45424 (14)	0.0356 (7)	
C5	0.5770 (4)	0.45689 (19)	0.41716 (13)	0.0303 (6)	
H5	0.5164	0.4058	0.4500	0.036*	
C6	0.4748 (4)	0.4443 (2)	0.34489 (14)	0.0382 (7)	
H6A	0.3540	0.4762	0.3490	0.046*	
H6B	0.5406	0.4823	0.3070	0.046*	
C7	0.4570 (4)	0.3248 (2)	0.32477 (15)	0.0393 (7)	
H7A	0.3784	0.2894	0.3599	0.047*	
H7B	0.3987	0.3192	0.2777	0.047*	
C8	0.6396 (4)	0.26588 (19)	0.32227 (13)	0.0319 (7)	
C9	0.7584 (4)	0.29023 (19)	0.38987 (14)	0.0319 (7)	
H9	0.6941	0.2567	0.4308	0.038*	
C10	0.7746 (3)	0.4128 (2)	0.40951 (13)	0.0304 (7)	
C11	0.9430 (4)	0.2297 (2)	0.38417 (15)	0.0471 (8)	
H11A	1.0415	0.2820	0.3799	0.057*	
H11B	0.9630	0.1879	0.4280	0.057*	
C12	0.9455 (4)	0.1535 (2)	0.31781 (15)	0.0490 (8)	
H12	1.0595	0.1119	0.3158	0.059*	
C13	0.9223 (5)	0.2234 (2)	0.24987 (17)	0.0558 (9)	
H13A	1.0244	0.2733	0.2459	0.067*	
H13B	0.9220	0.1776	0.2071	0.067*	
C14	0.7470 (5)	0.2859 (2)	0.25353 (15)	0.0409 (7)	
C15	0.6065 (4)	0.1418 (2)	0.32132 (16)	0.0448 (8)	
H15A	0.5334	0.1217	0.3631	0.054*	
H15B	0.5382	0.1227	0.2780	0.054*	
C16	0.7825 (4)	0.0797 (2)	0.32255 (15)	0.0444 (8)	
C17	0.7944 (5)	−0.0274 (2)	0.32922 (14)	0.0656 (10)	
H17A	0.6887	−0.0688	0.3335	0.079*	
H17B	0.9084	−0.0608	0.3296	0.079*	
C18	0.6115 (5)	0.6678 (2)	0.40765 (14)	0.0515 (9)	
H18A	0.5673	0.7334	0.4294	0.062*	
H18B	0.5617	0.6608	0.3597	0.062*	
H18C	0.7426	0.6701	0.4049	0.062*	
C19	0.3481 (4)	0.5846 (2)	0.47237 (17)	0.0556 (9)	
H19A	0.3332	0.6468	0.5030	0.067*	
H19B	0.3037	0.5214	0.4971	0.067*	
H19C	0.2801	0.5948	0.4283	0.067*	
C20	0.8897 (4)	0.4746 (2)	0.35366 (15)	0.0452 (8)	
H20A	1.0031	0.4371	0.3460	0.054*	
H20B	0.9141	0.5463	0.3713	0.054*	
H20C	0.8239	0.4790	0.3087	0.054*	
H1O	0.683 (4)	0.667 (3)	0.6009 (9)	0.083 (13)*	

### Atomic displacement parameters (Å^2^)

**Table:**

	*U*^11^	*U*^22^	*U*^33^	*U*^12^	*U*^13^	*U*^23^
O1	0.0756 (17)	0.0435 (13)	0.0414 (12)	0.0026 (13)	−0.0094 (14)	−0.0140 (11)
O2	0.0858 (18)	0.0589 (13)	0.0310 (10)	0.0106 (14)	0.0045 (12)	0.0076 (10)
C1	0.0394 (17)	0.0402 (15)	0.0437 (16)	0.0031 (15)	−0.0115 (16)	0.0003 (14)
C2	0.054 (2)	0.0401 (16)	0.0406 (17)	−0.0047 (17)	−0.0182 (17)	−0.0040 (15)
C3	0.0534 (18)	0.0316 (15)	0.0284 (14)	−0.0059 (15)	0.0001 (15)	−0.0041 (13)
C4	0.0402 (17)	0.0325 (15)	0.0341 (15)	0.0031 (14)	−0.0042 (14)	−0.0044 (13)
C5	0.0330 (16)	0.0308 (14)	0.0271 (14)	0.0007 (13)	−0.0034 (13)	0.0004 (12)
C6	0.0349 (17)	0.0426 (17)	0.0371 (16)	0.0054 (14)	−0.0103 (14)	−0.0042 (13)
C7	0.0388 (17)	0.0425 (16)	0.0365 (15)	−0.0001 (15)	−0.0076 (16)	−0.0063 (14)
C8	0.0358 (17)	0.0312 (14)	0.0288 (14)	−0.0008 (14)	0.0002 (15)	−0.0014 (13)
C9	0.0344 (16)	0.0317 (14)	0.0296 (13)	0.0009 (14)	0.0030 (14)	0.0035 (12)
C10	0.0309 (16)	0.0306 (15)	0.0296 (14)	−0.0024 (13)	−0.0024 (14)	0.0013 (12)
C11	0.048 (2)	0.0395 (16)	0.0537 (19)	0.0073 (16)	−0.0091 (18)	−0.0038 (15)
C12	0.0517 (19)	0.0470 (18)	0.0482 (18)	0.0178 (17)	0.0026 (18)	−0.0026 (17)
C13	0.061 (2)	0.0540 (18)	0.0522 (19)	0.0075 (19)	0.018 (2)	0.0013 (17)
C14	0.056 (2)	0.0359 (15)	0.0313 (15)	−0.0021 (17)	0.0015 (17)	−0.0038 (14)
C15	0.059 (2)	0.0381 (17)	0.0373 (16)	−0.0056 (16)	−0.0015 (18)	−0.0065 (15)
C16	0.065 (2)	0.0371 (16)	0.0307 (14)	0.0047 (17)	−0.0060 (17)	−0.0055 (14)
C17	0.097 (3)	0.0467 (19)	0.0533 (19)	0.011 (2)	−0.014 (2)	−0.0070 (17)
C18	0.081 (2)	0.0318 (16)	0.0415 (17)	0.0057 (19)	−0.0087 (19)	0.0020 (14)
C19	0.050 (2)	0.057 (2)	0.060 (2)	0.0091 (18)	−0.0085 (19)	−0.0210 (18)
C20	0.0452 (19)	0.0425 (16)	0.0479 (18)	−0.0069 (17)	0.0072 (16)	−0.0002 (14)

### Geometric parameters (Å, °)

**Table:**

O1—C3	1.429 (3)	C9—C10	1.560 (3)
O1—H1O	0.813 (10)	C9—H9	0.9800
O2—C14	1.216 (3)	C10—C20	1.533 (3)
C1—C2	1.527 (3)	C11—C12	1.544 (4)
C1—C10	1.543 (3)	C11—H11A	0.9700
C1—H1A	0.9700	C11—H11B	0.9700
C1—H1B	0.9700	C12—C16	1.502 (4)
C2—C3	1.507 (4)	C12—C13	1.531 (4)
C2—H2A	0.9700	C12—H12	0.9800
C2—H2B	0.9700	C13—C14	1.497 (4)
C3—C4	1.531 (3)	C13—H13A	0.9700
C3—H3	0.9800	C13—H13B	0.9700
C4—C19	1.537 (4)	C15—C16	1.498 (4)
C4—C18	1.548 (3)	C15—H15A	0.9700
C4—C5	1.562 (3)	C15—H15B	0.9700
C5—C6	1.535 (3)	C16—C17	1.331 (3)
C5—C10	1.550 (3)	C17—H17A	0.9300
C5—H5	0.9800	C17—H17B	0.9300
C6—C7	1.526 (3)	C18—H18A	0.9600
C6—H6A	0.9700	C18—H18B	0.9600
C6—H6B	0.9700	C18—H18C	0.9600
C7—C8	1.521 (4)	C19—H19A	0.9600
C7—H7A	0.9700	C19—H19B	0.9600
C7—H7B	0.9700	C19—H19C	0.9600
C8—C14	1.511 (4)	C20—H20A	0.9600
C8—C9	1.549 (4)	C20—H20B	0.9600
C8—C15	1.550 (3)	C20—H20C	0.9600
C9—C11	1.545 (4)		
			
C3—O1—H1O	109 (2)	C20—C10—C5	113.4 (2)
C2—C1—C10	113.0 (2)	C1—C10—C5	108.1 (2)
C2—C1—H1A	109.0	C20—C10—C9	111.6 (2)
C10—C1—H1A	109.0	C1—C10—C9	107.8 (2)
C2—C1—H1B	109.0	C5—C10—C9	107.0 (2)
C10—C1—H1B	109.0	C12—C11—C9	111.0 (2)
H1A—C1—H1B	107.8	C12—C11—H11A	109.4
C3—C2—C1	110.5 (2)	C9—C11—H11A	109.4
C3—C2—H2A	109.6	C12—C11—H11B	109.4
C1—C2—H2A	109.6	C9—C11—H11B	109.4
C3—C2—H2B	109.6	H11A—C11—H11B	108.0
C1—C2—H2B	109.6	C16—C12—C13	107.6 (3)
H2A—C2—H2B	108.1	C16—C12—C11	108.3 (2)
O1—C3—C2	112.1 (2)	C13—C12—C11	107.6 (2)
O1—C3—C4	108.4 (2)	C16—C12—H12	111.1
C2—C3—C4	113.7 (2)	C13—C12—H12	111.1
O1—C3—H3	107.5	C11—C12—H12	111.1
C2—C3—H3	107.5	C14—C13—C12	110.4 (3)
C4—C3—H3	107.5	C14—C13—H13A	109.6
C3—C4—C19	107.7 (2)	C12—C13—H13A	109.6
C3—C4—C18	110.8 (2)	C14—C13—H13B	109.6
C19—C4—C18	107.5 (3)	C12—C13—H13B	109.6
C3—C4—C5	107.3 (2)	H13A—C13—H13B	108.1
C19—C4—C5	108.4 (2)	O2—C14—C13	122.8 (3)
C18—C4—C5	115.0 (2)	O2—C14—C8	123.6 (3)
C6—C5—C10	109.8 (2)	C13—C14—C8	113.5 (3)
C6—C5—C4	114.4 (2)	C16—C15—C8	111.8 (2)
C10—C5—C4	117.6 (2)	C16—C15—H15A	109.3
C6—C5—H5	104.5	C8—C15—H15A	109.3
C10—C5—H5	104.5	C16—C15—H15B	109.3
C4—C5—H5	104.5	C8—C15—H15B	109.3
C7—C6—C5	110.5 (2)	H15A—C15—H15B	107.9
C7—C6—H6A	109.5	C17—C16—C15	124.5 (3)
C5—C6—H6A	109.5	C17—C16—C12	123.8 (3)
C7—C6—H6B	109.5	C15—C16—C12	111.7 (2)
C5—C6—H6B	109.5	C16—C17—H17A	120.0
H6A—C6—H6B	108.1	C16—C17—H17B	120.0
C8—C7—C6	113.3 (2)	H17A—C17—H17B	120.0
C8—C7—H7A	108.9	C4—C18—H18A	109.5
C6—C7—H7A	108.9	C4—C18—H18B	109.5
C8—C7—H7B	108.9	H18A—C18—H18B	109.5
C6—C7—H7B	108.9	C4—C18—H18C	109.5
H7A—C7—H7B	107.7	H18A—C18—H18C	109.5
C14—C8—C7	113.8 (2)	H18B—C18—H18C	109.5
C14—C8—C9	110.6 (2)	C4—C19—H19A	109.5
C7—C8—C9	112.0 (2)	C4—C19—H19B	109.5
C14—C8—C15	103.5 (2)	H19A—C19—H19B	109.5
C7—C8—C15	109.6 (2)	C4—C19—H19C	109.5
C9—C8—C15	106.8 (2)	H19A—C19—H19C	109.5
C11—C9—C8	110.0 (2)	H19B—C19—H19C	109.5
C11—C9—C10	114.7 (2)	C10—C20—H20A	109.5
C8—C9—C10	114.7 (2)	C10—C20—H20B	109.5
C11—C9—H9	105.5	H20A—C20—H20B	109.5
C8—C9—H9	105.5	C10—C20—H20C	109.5
C10—C9—H9	105.5	H20A—C20—H20C	109.5
C20—C10—C1	108.7 (2)	H20B—C20—H20C	109.5
			
C10—C1—C2—C3	58.1 (3)	C4—C5—C10—C1	50.0 (3)
C1—C2—C3—O1	176.9 (2)	C6—C5—C10—C9	−61.1 (3)
C1—C2—C3—C4	−59.7 (3)	C4—C5—C10—C9	165.85 (19)
O1—C3—C4—C19	−64.3 (3)	C11—C9—C10—C20	58.7 (3)
C2—C3—C4—C19	170.4 (2)	C8—C9—C10—C20	−70.0 (3)
O1—C3—C4—C18	53.0 (3)	C11—C9—C10—C1	−60.6 (3)
C2—C3—C4—C18	−72.3 (3)	C8—C9—C10—C1	170.7 (2)
O1—C3—C4—C5	179.3 (2)	C11—C9—C10—C5	−176.7 (2)
C2—C3—C4—C5	53.9 (3)	C8—C9—C10—C5	54.6 (3)
C3—C4—C5—C6	178.3 (2)	C8—C9—C11—C12	−6.4 (3)
C19—C4—C5—C6	62.3 (3)	C10—C9—C11—C12	−137.4 (2)
C18—C4—C5—C6	−57.9 (3)	C9—C11—C12—C16	−54.4 (3)
C3—C4—C5—C10	−50.6 (3)	C9—C11—C12—C13	61.7 (3)
C19—C4—C5—C10	−166.6 (2)	C16—C12—C13—C14	57.9 (3)
C18—C4—C5—C10	73.1 (3)	C11—C12—C13—C14	−58.6 (3)
C10—C5—C6—C7	62.9 (3)	C12—C13—C14—O2	−175.6 (3)
C4—C5—C6—C7	−162.4 (2)	C12—C13—C14—C8	0.7 (3)
C5—C6—C7—C8	−55.2 (3)	C7—C8—C14—O2	−1.0 (4)
C6—C7—C8—C14	−79.2 (3)	C9—C8—C14—O2	−128.1 (3)
C6—C7—C8—C9	47.2 (3)	C15—C8—C14—O2	117.9 (3)
C6—C7—C8—C15	165.5 (2)	C7—C8—C14—C13	−177.2 (2)
C14—C8—C9—C11	−51.2 (3)	C9—C8—C14—C13	55.7 (3)
C7—C8—C9—C11	−179.3 (2)	C15—C8—C14—C13	−58.3 (3)
C15—C8—C9—C11	60.7 (3)	C14—C8—C15—C16	61.1 (3)
C14—C8—C9—C10	79.9 (3)	C7—C8—C15—C16	−177.2 (2)
C7—C8—C9—C10	−48.2 (3)	C9—C8—C15—C16	−55.6 (3)
C15—C8—C9—C10	−168.2 (2)	C8—C15—C16—C17	173.1 (3)
C2—C1—C10—C20	71.8 (3)	C8—C15—C16—C12	−5.0 (3)
C2—C1—C10—C5	−51.7 (3)	C13—C12—C16—C17	126.9 (3)
C2—C1—C10—C9	−167.0 (2)	C11—C12—C16—C17	−117.1 (3)
C6—C5—C10—C20	62.4 (3)	C13—C12—C16—C15	−55.0 (3)
C4—C5—C10—C20	−70.7 (3)	C11—C12—C16—C15	61.1 (3)
C6—C5—C10—C1	−177.0 (2)		

### Hydrogen-bond geometry (Å, °)

**Table:**

*D*—H···*A*	*D*—H	H···*A*	*D*···*A*	*D*—H···*A*
O1—H1O···O2^i^	0.81 (1)	2.11 (1)	2.922 (3)	178 (3)

Symmetry codes: (i) −*x*+3/2, −*y*+1, *z*+1/2.
